# Is e-cigarette advertising associated with e-cigarette use among young people? New survey evidence from Poland

**DOI:** 10.3389/fpubh.2024.1448011

**Published:** 2024-10-07

**Authors:** Beata Świątkowska, Radosław Zajdel, Łukasz Balwicki, Dorota Kaleta

**Affiliations:** ^1^Department of Hygiene and Epidemiology, Medical University of Łódź, Łódź, Poland; ^2^Department of Informatics in Business and Medicine, University of Łódź, Łódź, Poland; ^3^Department of Public Health and Social Medicine, Medical University of Gdańsk, Gdańsk, Poland

**Keywords:** adolescents, advertising, e-cigarettes, youth, promotional activities

## Abstract

**Objective:**

Young people are routinely exposed to e-cigarettes advertising. We examined the impact of e-cigarette advertising on e-cigarette use in a large representative sample of adolescents.

**Methods:**

Data came from cross-sectional sample of the nationwide study on the health effects of tobacco products called PolNicoYouth, which included adolescents aged 15–18 years (*N* = 7,498). Data were collected through a detailed questionnaire recommended by international health organizations for monitoring tobacco use by adolescents. Simple and multiple logistic regression analyzes were conducted, adjusting for sex, age, type of school, place of residence, smoking of traditional cigarettes and parental smoking. Frequencies and proportions for descriptive statistics, and adjusted odds ratios with 95% confidence intervals for logistic regression models were reported.

**Results:**

Approximately, 56% of interviewees had noticed some form of e-cigarettes advertising. Exposure to e-cigarette advertising was significantly associated with ever use of e-cigarettes (OR = 1.29; 95% CI: 1.09–1.53). Exposure to e-cigarette advertising via club/pub/disco was significantly associated with current e-cigarette use (OR = 1.58; 95% CI: 1.06–2.36). Adolescents who have ever used e-cigarettes were more likely than never users to report exposure to advertisements on club/pub/disco (OR = 1.57; 95% CI: 1.08–2.30) and internet (OR = 1.22; 95% CI: 1.01–1.47).

**Conclusion:**

Despite the applicable advertising restrictions, the majority of young people declared contact with e-cigarette advertising, which shows the urgent need for more global action. The internet and advertisements in clubs, pubs and discos seem to be the key places of exposure. These forms of exposure need to be urgently addressed given their clear link to e-cigarette use.

## Introduction

Tobacco smoking worldwide, including in Poland, is a significant epidemiological and social problem. Poland is supposed to be a cigarette-free country, i.e., with a smoking rate of less than 5 percent, by 2030, meanwhile the number of tobacco smokers, including alternative tobacco products and e-cigarettes has been increasing since 2021. More and more women and teenagers are turning to cigarettes. According to the most up-to-date epidemiological study of 2022, 28.8% of the adult population (27.1% of women and 30.8% of men) in Poland already smoked. Of this, as many as 22.9% of women and 26.5% of men declared daily smoking. There were significant differences in the prevalence of daily use of heated tobacco according to age – people from younger age groups were most likely to use the new products (1). The fact that the small percentage of people trying to quit smoking or those who succeeded in quitting is worrying. The market for tobacco products and accessories is constantly changing. The use of electronic devices or heated tobacco is contributing to an increase in the percentage of people using nicotine products (2). The tobacco industry, looking for alternatives to the declining cigarette market, has expanded its product portfolio, introducing new products such as e-cigarettes and heated tobacco. This has contributed to the emergence of new consumer groups for nicotine products, which are most popular among teenagers and young adults.

Young people are less aware of the health risks of e-cigarettes and are more likely to use them than adults. Polish youth have virtually unlimited access to e-cigarettes. The law prohibiting their sale to minors is not enforced because despite the ban on advertising and promotion of tobacco products, these products are available to young people, as evidenced by statistics of their use (3). These regulations need to be updated to adapt them to current challenges, e.g., limiting online advertising or their effective application to alternative tobacco products. There is also a lack of elementary education to warn against the disastrous consequences of addiction. The figures for young people are alarming – 60 percent of all teenagers and almost half of 15-year-olds initiated nicotine use. In recent years there has been a significant increase in the popularity of e-cigarettes especially among young people. Teens are now more likely to choose e-cigarettes over traditional cigarettes (1). In addition, the phenomenon of dual use of tobacco and e-cigarettes is also observed (3). Factors contributing to nicotine initiation include peer pressure (peers, school), availability of tobacco products and exposure to advertising of nicotine products (4). Although e-cigarette advertising is limited in Poland, it plays a significant role in this trend, shaping positive perceptions of e-cigarettes and may play an important role in initiating and sustaining e-cigarette use among young adults.

The prevalence of e-cigarette use among adolescents has increased dramatically worldwide, and there are serious health risks associated with this behavior (5). E-cigarette use among adolescents has harmful effects on many aspects of health (6–8). Despite existing legal regulations prohibiting the advertising of these products, little is known about the real impact of advertising these products to adolescents. This information is essential for establishing effective policies or interventions to reduce e-cigarette use among teens.

This article aims to analyze the problem of youth exposure to e-cigarette advertising and identify the links between exposure to advertising for these products and e-cigarette use among adolescents.

## Materials and methods

### Study design and population

The study was part of a nationwide study on the health effects of tobacco products, which involved almost 2% of the population of primary and secondary school students aged 15–18, financed by the National Health Program, the Ministry of Health in Poland. The analysis was based on a large cross-sectional study conducted in the first 2 months of 2020 among 15,225 students from 200 Polish upper secondary schools using a random, stratified selection of institutions. This study analyzes data for 7,498 young people who declared ever e-cigarettes smoking.

The study was approved by the National Institute of Public Health PZH—Bioethical Committee of the National Research Institute (Resolution No. 3/2019; 13/11/2019).

### Measures

The data necessary for the analysis was collected using an online questionnaire, with the prior consent of the participants, using the Computer-Assisted Web Interview tool, which increases the reliability of data collection and allows to avoid errors that may occur during self-coding or entering data using survey software. The Global Youth Tobacco Survey (GYTS) questionnaire recommended by the World Health Organization (WHO) and the Centers for Disease Control and Prevention (CDC) to monitor youth tobacco use was used (9).

Participants provided information on the following demographic variables: their sex (female, male); age (15–17 years; ≥18 years); type of school (grammar or vocational/technical) and residence (urban or rural). A variable for parental smoking was also taken into account in the analysis (neither of the parents smoke vs. either or both parents smoke). The participants were asked whether they had ever used cigarettes, if they did also about their current smoking habits. Information on smoking traditional cigarettes and e-cigarette use was separately collected. People who never smoked are people who answered “no” to the question: Have you ever tried traditional cigarettes, even once in life?. Those who answered “yes” to this question were categorized as ever smokers. Current smokers were reported to have smoked in the past 30 days. The same type of question was asked to report e-cigarettes behavior. We first asked if they had ever tried an e-cigarettes, using the following item: Have you ever tried e-cigarettes, even once in life? If the participant answered “yes,” we assessed current cigarette use with the question: Have you used e-cigarettes at least once in the last 30 days? Adolescents who reported any use in the past 30 days were considered current cigarette smokers.

To assess e-cigarette advertising exposure, participants were asked, “Have you seen an advertisement for e-cigarettes in the last 30 days?.” Answer options were yes/no. For respondents who indicated that they had seen or heard an advertisement for e-cigarettes type of exposure to e-cigarette advertising was measured by asking participants about the channels through which they had noticed any e-cigarette advertisements in the previous 30 days: shop, internet and club/pub/disco. Respondents who answered “yes” have been classified as those who were exposed to tobacco advertising. The reference group for advertising exposure was “no exposure.”

### Statistical methods

In the descriptive analysis, the numbers of each group and their structure indicators are given. An analysis of the significance of differences in the abundance of each subgroup was performed. Statistical correlation analysis was performed using logistic regression, with the odds ratio calculated as a weighted indicator, multivariate logistic regression model assessed the relationship between e-cigarette advertising and (1) ever e-cigarette use and (2) current e-cigarette use. The following covariates were included in models: gender, age group, type of high school, type of residence, smoking, and parental smoking. The analysis was performed using the Statistica 13.3 package.

## Results

Table 1 summarizes the key characteristics among study population, 7,498 young adult (53.5% men and 46.5% women). Approximately, 56% of interviewees had noticed some form of e-cigarettes Advertising. Among young men, 46.2% reported exposure to e-cigarette advertising This ratio was similar in the group of young women and amounted to 47.0%. In total, 75.5% of the interviewees were averaged 15–17 years, slightly more than half of them (53.0%) have not seen an advertisement for e-cigarettes. The highest proportion of respondents (56.0%) had attained vocational and technical education, 43.5% of these people declared that they were exposed to e-cigarette Advertising. Most of the respondents came from rural areas, but in all groups of residence the percentage of people who were not exposed to e-cigarette advertising was higher.

**Table 1:** Characteristics of study population by status of exposure to e-cigarettes advertising.

Variables *n*	Exposure to e-cigarettes advertising *n* (%)		*p* value
	No	Yes	
**Population (overall)**			
Male (4014)	2,161 (53.84)	1853 (46.16)	0.000
Female (3484)	1850 (53.10)	1,634 (46.90)	0.000
**Age (years)**			
15–17 (5658)	2,998 (52.99)	2,660 (47.01)	0.000
≥18 (1840)	1,013 (55.05)	827 (44.95)	0.000
**Type of school**			
Grammar (3280)	1,631 (49.73)	1,649 (50.27)	0.662
Vocational/technical (4164)	2,352 (56.48)	1812 (43.52)	0.000
**Place of residence (number of inhabitants)**			
Rural (3352)	1774 (52.92)	1,578 (47.08)	0.000
Cities <20th (1487)	819 (55.08)	668 (44.92)	0.000
Cities 20–99 (1219)	611 (50.12)	608 (49.88)	0.906
Cities 100–500 (817)	425 (52.02)	392 (47.98)	0.103
Cities >500 (292)	157 (53.77)	135 (46.23)	0.068
**Smoking (traditional cigarettes)**			
Never (2768)	1,360 (49.13)	1,408 (50.87)	0.195
Ever (2058)	1,019 (49.57)	1,039 (50.43)	0.581
Current (2672)	1,632 (61.08)	1,040 (38.92)	0.000
**Smoking (e-cigarettes)**			
Never (2839)	1,459 (51.39)	1,380 (48.61)	0.036
Ever (1666)	795 (47.72)	871 (52.28)	0.009
Current (2993)	1757 (58.70)	1,236 (41.30)	0.000
**Parental smoking**			
Traditional cigarettes			
No (3826)	1970 (51.49)	1856 (48.51)	0.009
Yes (3155)	1,694 (53.69)	1,461 (46.31)	0.000
**e-cigarettes**			
No (6367)	3,336 (52.40)	3,031 (47.60)	0.000
Yes (468)	222 (47.44)	246 (52.56)	0.117

About 27.5% of individuals reported ever smoking, including 36.0% current smokers. The majority of participants were current e-cigarettes users (40.0%); 38.0% of young people reported never e-cigarette use and 22.2% having ever used e-cigarettes, even once in life. Among current e-cigarette users, almost 60% were not exposed to advertising of these products, while among never e-cigarettes users 51.4were those who had no to deal with such exposure. In total, 45.2% of parents were smokers and 54.8% were non-smokers. Only less than 7% of parents used e-cigarettes, Among teenagers whose parents used e-cigarettes, almost 53% were exposed to e-cigarette advertising. Table 2 shows the results of the multivariate regression analysis of the association between exposure to e-cigarettes advertising and ever or current e-cigarette use. As seen exposure to e-cigarette advertising was significantly associated with ever use of e-cigarettes (OR = 1.29; 95% CI: 1.09–1.53) when adjusting for sex, age, type of school, place of residence, smoking of traditional cigarettes and parental smoking. Likewise, e-cigarette advertising exposure was associated with current use of e-cigarettes, although in this case the results were not statistically significant (OR = 1.12; 95% CI: 0.93–1.34). Men were more likely than women to report e-cigarette use, both among ever (OR = 2.07; 95% CI:1.74–2.48) and current e-cigarette smokers (OR = 2.44; 95% CI: 2.01–2.95). In both groups the risk was higher among grammar school students, OR = 1.71 (95% CI: 1.42–2.07) and 1.65 (95% CI: 1.35–2.02), respectively. For respondents who were living in the largest cities, the odds of ever e-cigarette use increased by 1.23, while the odds of current e-cigarette use amounted to OR = 2.28 (95% CI: 1.23–4.20). Individuals who additionally smoked traditional tobacco were over 2.5 times more likely to be ever user of e-cigarettes (OR = 2.60; 95% CI: 2.18–3.09) and more than four times were a current e-cigarette user (OR = 4.22; 95% CI: 3.51–5.08). We also assessed the differences in exposure through specific advertising channels. As shown in Table 3, internet was the most frequently reported source of advertising exposure among study participants. Other channels of exposure included shop and club/pub/disco. Logistic regression models showed that adolescents who have ever used e-cigarettes were more likely than never users to report exposure to advertisements on club/pub/disco (OR = 1.57; 95% CI: 1.08–2.30) and internet (OR = 1.22; 95% CI: 1.01–1.47). Compared to non-e-cigarette over 18 years of age users, younger (from 15 to 17 years of age) users of e-cigarettes were more likely to report e-cigarette use (OR = 1.20; 95% CI: 1.03–1.41). In addition, ever e-cigarettes smoking grammar school students were almost twice (OR = 1.81; 95% CI: 1.52–2.15) more likely to report exposure to e-cigarettes compared to vocational/technical students who had never tried e-cigarettes. A significantly higher risk was found among men with the relative risk being over two times greater in men than women (OR = 2.21; 95% CI: 1.88–2.59). This risk depended also on the place of residence. Thus, for respondents who were lived in big cities to the odds of e-cigarette use increased by 2.25 (OR = 2.25; 95% CI: 1.25–4.06). In the case of additional smoking traditional cigarettes, the risk increases more than 2.5 times (OR = 2.69; 95% CI: 2.29–3.17).

**Table 2:** Association between e-cigarette use and exposure to e-cigarettes advertising.

Variables	Ever e-cigarette use	Current e-cigarette use
	Adjusted OR (95%CI)	Adjusted OR (95%CI)
**Exposure to e-cigarettes advertising**		
No (ref)	–	–
Yes	1.29 (1.09–1.53)	1.12 (0.93–1.34)
Male	2.07 (1.74–2.48)	2.44 (2.01–2.95)
Female (ref)	–	–
**Age (years)**		
15–17	1.11 (0.93–1.34)	1.18 (0.97–1.44)
≥18 (ref)	–	–
**Type of school**		
Grammar	1.71 (1.42–2.07)	1.65 (1.35–2.02)
Vocational/technical (ref)	–	–
Place of residence (number of inhabitants)		
Rural (ref)	–	–
Cities <20th	1.36 (1.09–1.70)	1.47 (1.16–1.86)
Cities 20–99th	1.55 (1.21–1.98)	1.80 (1.38–2.34)
Cities 100–500	1.41 (1.05–1.84)	1.62 (1.19–2.22)
Cities >500	2.23 (1.24–4.02)	2.28 (1.23–4.20)
**Current traditional cigarette use**		
No (ref)	–	–
Yes	2.60 (2.18–3.09)	4.22 (3.51–5.08)
**Parental smoking**		
No (ref)	–	–
Yes	1.02 (0.86–1.21)	1.08 (0.90–1.30)

**Table 3:** Association between e-cigarette use and exposure to e-cigarettes advertising, by source of exposure.

Variables	Ever e-cigarette use	Current e-cigarette use
	Adjusted OR (95%CI)	Adjusted OR (95%CI)
**Exposure to e-cigarettes advertising**		
No (ref)	–	–
Shop	1.14 (0.90–1.44)	0.97 (0.75–1.24)
Internet	1.22 (1.01–1.47)	0.95 (0.78–1.16)
Club/pub/disco	1.57 (1.08–2.30)	1.58 (1.06–2.36)
Male	2.21 (1.88–2.59)	1.99 (1.68–2.36)
Female (ref)	–	–
**Age (years)**		
15–17	1.20 (1.03–1.41)	0.94 (0.79–1.12)
≥18 (ref)	–	–
**Type of school**		
Grammar	1.81 (1.52–2.15)	1.36 (1.13–1.64)
Vocational/technical (ref)	–	–
**Place of residence (number of inhabitants)**		
Rural (ref)	–	–
Cities <20th	1.42 (1.14–1.76)	1.29 (1.03–1.63)
Cities 20–99th	1.60 (1.26–2.05)	1.59 (1.23–2.06)
Cities 100–500	1.45 (1.09–1.94)	1.43 (1.05–1.94)
Cities >500	2.25 (1.25–4.06)	2.02 (1.10–3.69)
**Current traditional cigarette use**		
No (ref)	–	–
Yes	2.69 (2.29–3.17)	3.58 (3.01–4.26)
**Parental smoking**		
No (ref)	–	–
Yes	1.06 (0.90–1.25)	0.92 (0.78–1.10)

As seen Table 3 exposure to e-cigarette advertising via club/pub/disco was significantly associated with current e-cigarette use among young people when adjusting for sex, age, type of school, place of residence, current tobacco use and parental smoking (OR = 1.58; 95% CI: 1.06–2.36). Further, for additional traditional tobacco smoking students’ odds of current e-cigarette use increased by 3.58 (OR = 3.58; 95% CI: 3.01–4.26). Again, for participants who were living in big cities the odds ratio of current e-cigarette use increased by 2.02 (95% CI: 1.10–3.69). A lower level of education among current e-cigarette smokers was associated with a higher risk of e-cigarette use (OR = 1.36; 95% CI: 1.13–1.64) compared to youth with vocational/technical education. Men who were current e-cigarette users, had a 2-fold (OR = 1.99; 95% CI: 1.68–2.36) higher risk of e-cigarette use compared to women, who have never used e-cigarettes.

## Discussion

The use of a ban on all forms of advertising, promotion and sponsorship of e-cigarettes is one of the main strategies to reduce e-cigarette use among minors and non-smokers introduced by the World Health Organization (5) and this is in line with Article 13 of the Framework Convention on Tobacco Control (10). However, regardless of the regulatory framework in place, young people routinely encounter e-cigarette advertising. The results of our study will contribute to providing scientific evidence assessing the impact of e-cigarette advertising and marketing on e-cigarette use among young people. Regulations prohibiting marketing activities, including e-cigarette advertising, are a key factor in reducing the harms associated with e-cigarette use. Meanwhile, the vast majority (56%), of teenagers, participants in this study, were exposed to e-cigarette advertising in at least one type of media. Exposure rates were particularly high for the Internet. These are alarming data and unfortunately confirm data from other countries (11–13).

Our findings suggest that exposure to e-cigarette advertising was associated with e-cigarette use among young people in Poland. These relationships apply to ever e-cigarette use as well as current e-cigarette use. We further found that later e-cigarette use was related to various e-cigarette-related advertising channels. This is consistent with previous research on the relationship between exposure to e-cigarette advertising and e-cigarette use also. There is limited research on the impact of marketing on the use of e-cigarettes. These studies have found an association between exposure to tobacco product marketing and with increased likelihood of ever and current e-cigarette use (14–16). Exposure has also been linked to susceptibility to e-cigarette use among those who do not currently use them and as the number of channels of exposure to e-cigarette marketing increased, so did the likelihood of use and susceptibility (17).

E-cigarettes are not a safe substitute for tobacco products (6, 18). A recent large review of 38 studies found that current e-cigarette use is associated with significantly lower quit rates among smokers and smokers are 28% less likely to quit using e-cigarettes than without them (19). According to recent studies, e-cigarettes are not at all associated with any change in the use of traditional cigarettes. Not only are e-cigarettes ineffective as a smoking cessation aid, but they actually promote – especially in adolescents – a descent into nicotine addiction (20, 21). Weak enforcement of the ban on point-of-sale advertising of tobacco products and e-cigarettes gives the tobacco industry a chance to promote its products illegally (22, 23). There is also a lack of prevention messages about e-cigarette use coming from the family, educational and social spheres (24, 25). Young adults are sceptical of the available scientific data on e-cigarette use and by choosing e-cigarettes over cigarettes, young adults believe they are making an informed and healthier choice. Recent results showed that 52.2 and 61.9% of young people, respectively, perceive e-cigarettes and heated tobacco products as less harmful compared to traditional cigarettes. The highest percentage of those who rated these products as less harmful was among current tobacco smokers (69.1%) (26).

Despite a large study group and a standardized survey instrument, our study has some limitations. First, the relationship between frequency of exposure to advertising and the type of advertising messages used and the risk of e-cigarette use was not examined in the study. It is possible that more frequent exposure to marketing activities in this area may be more strongly associated with e-cigarette use. Secondly, we examined the exposure of e-cigarette advertising through the most popular channels such as shop, internet and club/pub/disco. This is related to the introduced restriction on tobacco product advertising, no less advertising may also be related to other channels that may have a potential impact on e-cigarette use among young people. Thirdly, although the survey is representative, it concerns a population of young Poles in whom the problem of e-cigarettes and heated tobacco has emerged relatively recently. In addition, the measures of exposure to e-cigarette advertising and use were self-reported exposures. And we used e-cigarette use in the past 30 days as the outcome variable, which did not fully reflect the intensity of e-cigarette use among young people.

Despite the limitations indicated, the results of our analysis have significant merit indicating the problem of high prevalence of e-cigarette advertising among young people and the factors associated with this relationship. In order to answer the question whether exposure to e-cigarette advertising leads to e-cigarette use, a prospective study is required as a further direction for research. Future research can build on our study and be conducted more broadly, ignoring our limitations.

## Conclusion

Despite the advertising restrictions in place, the vast majority of young people said they had been in contact with e-cigarette advertising. The Internet and advertisements for pubs clubs, discos appear to be key exposure sites. These forms of exposure need to be urgently addressed, given their clear connection to e-cigarette use.

E-cigarettes should in any case be considered unhealthy and the arguments about the benefits of electronic cigarettes are unfounded, so their use, especially among young people, should be strongly restricted. As a result of the nicotine industry’s marketing efforts, young people are more likely to use e-cigarettes, which can lead to their addiction. The marketing of e-cigarettes is particularly geared toward reaching young people. Advertising of e-cigarettes can make young people see their use as normal and acceptable. Advertisements often portray e-cigarettes as trendy and attractive, which can encourage young people to try them. Advertisements also often overlook or minimize the risks associated with e-cigarette use which can lead to misconceptions about their safety.

Given the ever-increasing importance of e-cigarette use by young people as a global health problem, strengthening prevention strategies including the introduction of stricter restrictions and regulations on e-cigarette marketing and advertising and compliance is key. To reduce exposure to and access to e-cigarettes, the ubiquity of e-cigarette advertising and the persistent challenges of e-cigarette enforcement must be addressed.

## Data Availability

The datasets presented in this article are not readily available because the original contributions presented in the study are included in the article. Requests to access the datasets should be directed to the corresponding author.
